# Rehabilitation on the day of cardio-aortic surgery

**DOI:** 10.1186/1749-8090-10-S1-A79

**Published:** 2015-12-16

**Authors:** Hiroyuki Irie, Masahiko Ikebuchi, Hideki Teshima, Yusuke Kinugasa, Yosuke Miyamoto, Toshikazu Sano

**Affiliations:** 1 Department of Cardiovascular Surgery, Chikamori Hospital Heart Center, Kochi, Japan

## Background/Introduction

Fast-track strategy in cardio-aortic surgery has been known to have advantages in minimizing post-operative complications, shortening hospital stay and lowering costs.

## Aims/Objectives

We reviewed our rehabilitation plan starting 2 hours after cardio-aortic operations retrospectively, in terms of feasibility and safety.

## Method

Inclusion Criteria: 1. Non-emergency surgery; CABG, valve surgery, thoracic aortic surgery and their combination. 2. Well-awake without a ventilator 2 hours after operation. 3. No femoral lines such as IABP or arterial lines. 4. Chest tube drainage less than 100 ml/h. 5. No neurological, hemodynamic, or subjective abnormalities.

During two years, 281 patients met the criteria and divided into two groups: Group 0 consisting of 160 patients started rehabilitation (124 standing and 36 sitting) on the day of operation and Group 1 of 121 patients on post-operative day 1. Statistical analysis was considered significant when p-value was less than 0.05.

## Results

Both groups had no significant difference in height, body weight, pre-op EF, duration of anesthesia or aortic cross-clamp time. Group 1, however, had significantly higher values in age (70.0 ± 11.0 vs 73.2 ± 12.1 years), EuroSCORE II (2.3 ± 1.7 vs 5.2 ± 5.5), operation time (261 ± 60 vs 327 ± 115 min), water balance (446 ± 1037 vs 936 ± 1268 ml), days requiring to achieve pre-operative walkiAng level (4.2 ± 4.7 vs 7.5 ± 8.2), post-operative hospital stay (13 ± 12.8 vs 23 ± 32 days) and in-hospital mortality (0.6 vs 5.0%). There was no adverse event due to rehabilitation during the study period.

## Discussion/Conclusion

In summary, rehabilitation 2 hours after operations was safe. Group 0 showed faster recovery, but had younger age and lower operative risks. Further analysis should be considered to clarify that early intervention could be beneficial to which patients and to what extent.

